# Genotyping of Porcine Circovirus 2 (PCV-2) in Vaccinated Pigs Suffering from PCV-2-Systemic Disease between 2009 and 2020 in Spain

**DOI:** 10.3390/pathogens10081016

**Published:** 2021-08-12

**Authors:** Marina Sibila, Caterina Rocco, Giovanni Franzo, Eva Huerta, Mariano Domingo, José Ignacio Núñez, Joaquim Segalés

**Affiliations:** 1 IRTA, Centre de Recerca en Sanitat Animal (CReSA, IRTA-UAB), Campus de la Universitat Autònoma de Barcelona, Bellaterra, 08193 Barcelona, Spain; crocco.p89@gmail.com (C.R.); eva.huerta@irta.cat (E.H.); joseignacio.nunez@irta.cat (J.I.N.); 2 OIE Collaborating Centre for the Research and Control of Emerging and Re-Emerging Swine Diseases in Europe (IRTA-CReSA), Bellaterra, 08193 Barcelona, Spain; mariano.domingo@irta.cat (M.D.); joaquim.segales@irta.cat (J.S.); 3 Department of Animal Medicine, Production and Health (MAPS), University of Padua, 35020 Padua, Italy; giovanni.franzo@unipd.it; 4 Departament de Sanitat i Anatomia Animals, Universitat Autònoma de Barcelona, Bellaterra, 08193 Barcelona, Spain; 5 UAB, Centre de Recerca en Sanitat Animal (CReSA, IRTA-UAB), Campus de la Universitat Autònoma de Barcelona, Bellaterra, 08193 Barcelona, Spain

**Keywords:** porcine circovirus 2, genotypes, PCV-2-SD, vaccination

## Abstract

Vaccination against porcine circovirus 2 (PCV-2) is a common practice all over the world. Vaccines can prevent PCV-2-systemic disease (PCV-2-SD) outbreaks but not PCV-2 infection, which can be detectable in a percentage of vaccinated animals. Occasionally, PCV-2-SD is diagnosed in vaccinated farms. The objective of this study was to genotype the PCV-2 strains detected in vaccinated animals diagnosed with PCV-2-SD. Additionally, the evolution of the frequency of PCV-2 genotype detection at Spanish, European, and world levels was assessed. Fifty cases diagnosed as PCV-2-SD between 2009 and 2020 were included in this study. PCV-2 genotype was determined by sequencing the Cap gene region. Among them, only PCV-2b (23/50, 46%) and PCV-2d (27/50, 54%) genotypes were detected. Although the frequency of detection of these two genotypes was similar, their temporal distribution was different. Whereas most PCV-2b sequences (17/23, 74%) were detected between 2009 and 2012, PCV-2d sequences were obtained from 2013 to 2020. Indeed, a predominance of the PCV-2d genotype was observed from 2013 onwards, a trend also noticed at European and world levels. The results suggest that detection of particular genotypes in vaccinated animals probably reflects the general prevalence of the genotypes over time rather than genotype-specific vaccine-immunity escaping.

## 1. Introduction

Porcine circovirus 2 (PCV-2) is the main causative agent of a group of diseases known as porcine circovirus associated diseases (PCVD) which include PCV-2 systemic disease (PCV-2-SD), porcine dermatitis and nephropathy (PDNS), PCV-2 reproductive disease (PCV-2-RD), and finally PCV-2 subclinical infection (PCV-2-SI) [1]. PCV-2-SD and PCV-2-SI are the most economically important among them [2]. Although vaccination against PCV-2 has drastically reduced the impact of PCVDs, diagnosis of PCV-2-SD is still established occasionally. Diagnostic criteria for PCV-2-SD include the presence of clinical signs compatible with the disease (growth retardation, wasting, and respiratory and/or digestive clinical signs) and the observation of characteristic histologic lesions in lymphoid tissues (lymphocyte depletion [LD], histiocytic infiltration [HI], and presence of multinucleated cells and/or intracytoplasmic inclusions), together with the detection of moderate to marked amount of PCV-2 in damaged tissues [3]. 

So far, and based on ORF2 sequencing, up to nine different PCV-2 genotypes have been described [4,5,6]. Among them, PCV-2a, PCV-2b, and PCV-2d show a global distribution and are considered the most prevalent ones [6]. The remaining ones have been detected much more sporadically and in very limited areas [4]. Nevertheless, the PCV-2 genotype global prevalence is considered rather dynamic. In 1996, the most frequent genotype in pigs was PCV-2a, but in 2000–2004, a global genotype shift from PCV-2a to PCV-2b occurred [7,8,9,10]. This first genotype prevalence change took place when no PCV-2 vaccination was available and was associated with more severe clinical disease outbreaks. Some years later (2010 onwards), PCV-2b was slowly replaced by PCV-2d [11,12,13]. This latter genotype has been proposed as a potential cause of vaccination failures [14] but also suggested as a genotype with increased virulence [15]. However, subsequent experimental studies have shown that PCV-2d does not escape the immunity conferred by vaccines based on the PCV-2a genotype [14,16], and its virulence is not different from that of PCV-2a and PCV-2b [17]. 

Vaccination of piglets and/or gilts/sows against PCV-2 is a common practice all over the world. Most of these vaccines are based on PCV-2a genotypes. PCV-2 vaccines can prevent clinical disease (PCV-2-SD) but not infection, which is still widespread throughout the vaccinated population [18]. However, PCV-2-SD is occasionally diagnosed on vaccinated farms. Therefore, the topic of lower protection induced by current vaccines against PCV-2d under field conditions is still a matter of debate among field veterinarians.

The present work aimed to assess the PCV-2 genotypes associated with PCV-2-SD cases diagnosed in vaccinated animals in Spain during the period 2009–2020. In addition, the PCV-2 genotype evolution was analyzed from world, European, and Spanish perspectives.

## 2. Results

### 2.1. PCV-2 Phylogenetic Analyses and Genotyping 

**Figure 1:**
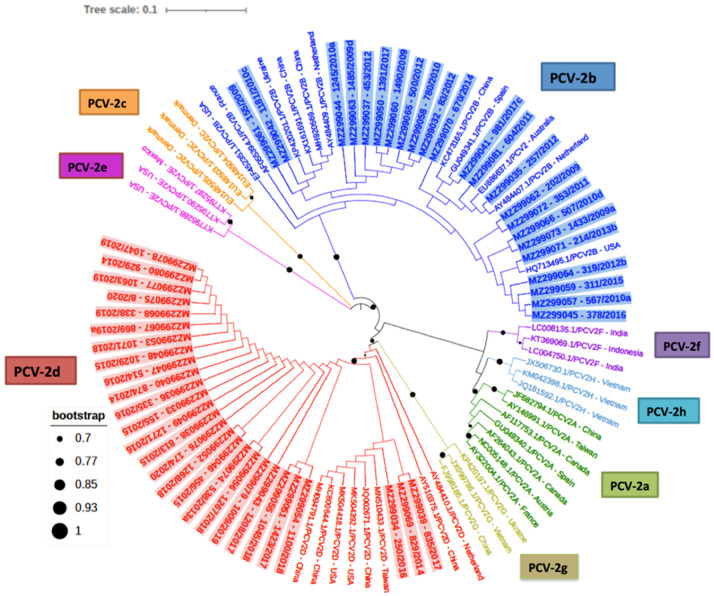
Phylogenetic tree inferred using the maximum likelihood method based on 91 nucleotide PCV-2 sequences including the 50 samples obtained in this study (in bold and shaded in blue for PCV-2b and in red for the PCV-2d plus the sample number and year) and 41 strains of the different genotypes available in GenBank (PCV-2a to PCV-2h) (no bold or shading and identified with the country of origin). Black circles in branches represent bootstrap values. Circle sizes are proportional to the bootstrap value (only values higher than 0.70 are shown).

Among the 50 PCV-2 ORF2 sequences obtained, only PCV-2b and PCV-2d genotypes were detected and showed similar frequencies (23/50 [46%] and 27/50 [54%], respectively) (Figure 1). The percentage of identity among the PCV-2b genotype sequences was between 98.1% and 100% and between 99.0% and 100% for PCV-2d strains. None of the studied samples yielded more than one genotype.

Although the frequency of detection of these two genotypes was quite similar, their yearly distribution was fairly different. Whereas PCV-2b was firstly detected in 2009, PCV-2d was identified for the first time in 2013 (Table 1). Indeed, in the period 2009–2012, only PCV-2b was detected (17/17, 100%), while from 2013 to 2017, both genotypes co-existed (PCV-2b 6/21 [29%] and PCV-2d 15/21, [71%]), and finally, from 2018 to 2020, only PCV-2d (12/12, 100%) was found.

### 2.2. PCV-2 Genotype Frequencies at the World, European, and Spanish Levels 

**Figure 2:**
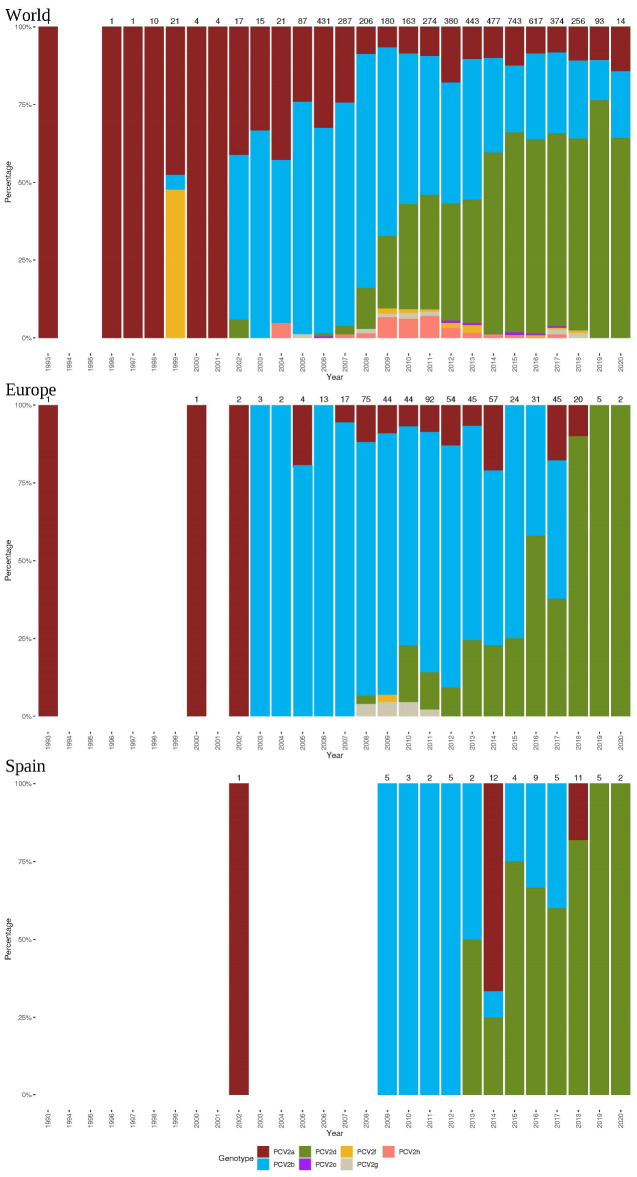
PCV-2 genotype frequency pattern at world, European, and Spanish levels. The proportion (in percentage) of each genotype (color coded) is plotted against time. The raw total sequence number is reported for each column. Years with no bar mean lack of available sequences.

A total of 5120, 581, and 66 sequences with available collection date and country were included in the world, European, and Spanish datasets. While in Spain, only genotypes PCV-2a, b, and d were detected, PCV-2f and PCV-2g were reported in other European countries but extremely sporadically (Figure 2). Genotype frequency evaluation over time highlighted a common pattern among different epidemiological scales. Overall, PCV-2a and PCV-2b predominated in the 1990s and the first decade of the new millennium, respectively. From that moment onwards, the rise in PCV-2d detection frequency compared to other genotypes was evident at the Spanish, European, and world levels. 

## 3. Discussion

In the present study, the PCV-2 genotype detected in 50 vaccinated animals that suffered from PCV-2-SD was determined by sequencing their ORF2 sequence. The reason behind the development of this pathology was not herein investigated. Indeed, it is unknown if the PCV-2-SD development was associated with a vaccine failure, wrong vaccine application, wrong vaccination timing, or vaccination of pre-infected animals.

Results obtained in this study evidenced that the PCV-2-SD cases diagnosed between 2009 and 2020 in PCV-2 vaccinated farms were associated either with PCV-2b or PCV-2d genotypes. Although the overall frequency of detection of these two genotypes in this time frame was quite similar, their temporal distribution was different. Whereas PCV-2b was found from 2009 to 2017, PCV-2d was reported from 2013 to 2020. This tendency of PCV-2b replacement by PCV-2d is endorsed by the results obtained in other pig-producing countries where a rise of PCV-2d detection in recent years has been detected. For example, Hou et al. (2019) reported an increase in PCV-2d frequency from 54.5 to 71.4% between 2016 and 2018 in tissue samples from pigs coming from 65 different Chinese farms [19]. In a study conducted in Taiwan during 2016 and 2017 where tissue samples from 214 PCVD cases were analyzed, a predominance of PCV-2d over PCV-2b after 2010 was also observed [13]. Moreover, in a study performed in Austria, although PCV-2b was the most prevalent genotype detected in the tissue samples tested from 2002 to 2017, an increase of PVC-2d (from 10% in 2015 to 70% in 2017) was also noticed [20]. Recently, PCV-2d has been detected for the first time in Australia, albeit PCV-2b is still predominant there [21]. 

In most of these studies, the vaccination status of the animals was not reported. However, given the wide use of PCV-2 vaccines across pig producing countries [19,22], it can be assumed that most of them were under the frame of PCV-2 vaccination. The expansion of PCV-2d over the other genotypes in a global vaccinated population may suggest that this rise in frequency could be linked to farm vaccination status. In fact, this genotype has been described as more virulent and a potential immunological escape mutant [14,15]. Nevertheless, this hypothesis is currently not supported by experimental evidence [17,23]. The current major prevalence of the PCV-2d genotype all over the world is most likely due to a higher viral fitness than other genotypes under the current vaccination scenario. Indeed, it has been proposed that viral evolution exerted by natural immunity and vaccine-acquired immunity has favored mutations towards a better receptor-binding capacity of the Cap protein with the cellular receptor [24]. In fact, it has been suggested that vaccine-induced immune-escaping evolutive trajectories have already occurred for PCV-2 [25] and may appear again in the future. Whether currently used vaccines are more effective on PCV-2b or whether a fast-evolving virus such as PCV-2 simply produced a novel genotype (PCV-2d) with better biological fitness is not yet known [26]. Nevertheless, the detection of both PCV-2b and PCV-2d in vaccinated animals with comparable frequency herein reported suggests that these evolutive forces could have had an effect at the epidemiological level but are unlikely to be significant in terms of cross-protection and clinical impact. 

Therefore, based on present results in PCV-2-SD cases from 2009 to 2020 in Spain, it is very likely that genotype detection in vaccinated animals simply reflected the major genotype prevalence during that period rather than a potential vaccine failure. The Spanish, European, and worldwide available sequences analyzed in the present study further confirm this hypothesis, since an evident trend of higher frequencies of PCV-2a during the 1990s, PCV-2b during the 2000s, and PCV-2d from 2010 to 2012 onwards was observed. Such results are not surprising since other studies already pointed out such evolution over time [11,12]. 

Although the results of the present study can be somewhat biased by the fact that the cases examined belonged to diseased pigs submitted to a diagnosis service, the information provided can be very useful to monitor the constant PCV-2 genotype evolution [27].

## 4. Materials and Methods

### 4.1. Sample Selection 

In this study, 50 animals submitted to the *Servei de Diagnòstic de Patologia Veterinària* at the Veterinary School of Universitat Autònoma de Barcelona (Spain) between 2009 and 2020 were retrospectively selected because of being diagnosed as unequivocal PCV-2-SD cases [1] and coming from 50 PCV-2 vaccinated farms. In all farms, vaccination against PCV-2 was performed in pigs around the age of weaning (3–4 weeks of age). Affected farms were from all over the Spanish geography, but most of them were placed in the northeastern part, where the high-density pig rearing areas are located.

These animals were submitted for diagnosis due to significant problems in their respective herds in terms of clinical signs compatible with the disease [28]. The age of the animals varied between 3 and 16 weeks (end of nursery and fattening pigs). At necropsy, tonsil and tracheo-bronchial, mesenteric, and inguinal superficial lymph nodes were collected and immersed in formalin. Once in the lab, fixed tissues were dehydrated and embedded in paraffin. From each paraffin block, different slices were made. One section was stained with hematoxylin and eosin and used for assessing the presence of lesions compatible with PCV-2-SD (LD and HI) and another one for assessing the presence of PCV-2 by immunohistochemistry (IHC). 

All animals included in this retrospective study showed moderate to severe LD together with HI of lymphoid tissues and had moderate to high amount of PCV-2 antigen. Consequently, they were individually diagnosed as PCV-2-SD [29]. The number of PCV-2-SD diagnosed and tested cases per year varied from 2 to 5. 

### 4.2. Deparaffination and DNA Extraction Protocol 

Four tissue slices of 8 µm thickness were immersed in 1 mL of Xylol, centrifuged at maximum speed (20,000× *g*) for 2 minutes, and the supernatant was removed. These steps were repeated 2 more times. After these deparaffination steps, the pellet was resuspended with absolute ethanol two times. Subsequently, and in order to remove ethanol, the pellet was dried at 37 °C for 1 h. Finally, DNA was extracted using the QiAamp^®^ DNA FFPE Tissue kit (Qiagen, Hilden, Germany), according to manufacturer’s instructions. 

### 4.3. PCV-2 CAP Gene Amplification and Sequencing 

PCV-2 genotype was determined by amplifying and sequencing the PCV-2 capsid protein gene (ORF2). This gene was amplified using a previously described procedure [30] but using a modified version of the reverse primer (5′-CGTATCCAAGGAGGCGTTAC-3′). The PCR was carried out using the GoTaq^®^ G2 Flexi DNA Polymerase Kit (Promega, Madison, MI, USA) containing 2.5 μL of the extracted 1/100 diluted DNA, 1.25 μL of each primer at 10 pmol/μL, 5 μL of 5 × Green GoTaq^®^ Flexi Buffer, 2.5 μL of MgCl_2_ at 25 mM, 0.15 U GoTaq^®^ polymerase (5U/μL), 1 μL of dNTP at 5 mM, and up to 25 μL of DEPC-treated water. The amplification parameters were as follows: initial denaturation of 5 min at 94 °C, followed by 35 cycles of 95 °C for 30 s, 53 °C for 30 s, and 72 °C for 40 s, with a final elongation at 72 °C for 7 min. Subsequently, the amplified PCR product was visualized through electrophoreses in a 2% agarose gel. 

The PCR amplicon was purified using the NucleoSpin Gel^®^ and PCR Clean-up kit (Macherey-Nagel, Düren, Germany), according to the manufacturer’s instructions. The quality and quantity of DNA from each sample were analyzed with Biodrop (Biodrop Ltda, Cambridge, UK) and then submitted to Servei de Genòmica i Bioinformàtica of the Universitat Autònoma de Barcelona (Spain) for Sanger DNA sequencing with the ABI PRISM sequencer 3130xl (Applied Biosystem^®^, Waltham, MA, USA). 

### 4.4. PCV-2 ORF2 Phylogenetic Analyses

The sequence quality evaluation and the assembly of the consensus sequences was carried out using the program ChromasPro version 2.1.8 (Technelysium, South Brisbane, QLD, Australia). The sequences were aligned, together with 41 strains of different PCV-2 genotypes recovered from the GenBank database, using the Clustal Omega multiple alignment method (https://www.ebi.ac.uk/Tools/msa/clustalo accessed on 22 February 2021). The phylogenetic tree was constructed according to the maximum likelihood method and the Kimura-2 parameter model [31] using the MEGA version X program (Mega Limited, Auckland, New Zealand) [32] with 1000 bootstrap replicates. The tree was edited with the online program ITOL (https://itol.embl.de, accessed on 9 July 2021). The distance matrices were calculated from each data set using the Clustal Omega program. The percentage of identity among PCV-2b and PCV-2d sequences was calculated using BLAST (https://blast.ncbi.nlm.nih.gov/Blast.cgi, accessed on 28 May 2021). 

The consensus sequences of PCV-2 ORF2 were deposited at the NCBI GenBank with the numbers (MZ299032–MZ299081).

### 4.5. Phylogenetic Analyses of PCV-2 Sequences at World, European, and Spanish Levels

All PCV2 ORF2 sequences were downloaded from GenBank. Relative metadata, including collection country and date, were extracted using in-house developed Python scripts. Spanish sequences were added to the dataset, and a preliminary alignment was performed at the amino acid level and then backtranslated at the nucleotide one using TranslatorX.

All poorly aligned sequences, those displaying premature stop codons or frameshift mutations, were removed from the alignment. After refinement, a new alignment was performed at the amino acid level and then backtranslated at the nucleotide one using TranslatorX and scanned for recombinant sequences removal using RDP5. RDP, GENECONV, Chimaera, and 3Seq were selected for the primary scan, while the whole set of available methods was used for recombination confirmation. The method’s settings were adjusted based on the dataset features, and recombination events were accepted only if detected by more than two methods with a significance level of p < 0.001 with Bonferroni correction. Finally, sequences were aligned with the reference dataset provided by [4]. To maintain the maximum sequence number, still preserving classification robustness, a partial ORF2 alignment was created, encompassing a region of 495 nucleotides. The consistency with the original classification approach was confirmed by analyzing the corresponding region of the reference dataset and assessing the absence of significant differences in strain classification. 

A phylogenetic tree was reconstructed in MEGAX for genotype classification purpose. The robustness of inferred clades was assessed by performing 10,000 bootstrap replicates. 

## 5. Conclusions

In conclusion, PCV-2 showed a dynamic and time-dependent genotype distribution between 2009 and 2020 in Spain, PCV-2b being the most frequently found genotype in early PCV-2-SD cases in vaccinated farms, and PCV-2d in subsequent ones. Such temporal genotype distribution apparently reflects the epidemiological evolution and spread of the genotypes over time rather than vaccine-immunity escape outbreaks.

## Figures and Tables

**Table 1:** PCV-2 genotype yearly distribution among the 50 PCV-2-SD cases analyzed (in parentheses, percentage of each genotype within a year).

Year	N	Genotypes	
		PCV-2b (%)	PCV-2d (%)
2009	5	5 (100)	0 (0)
2010	5	5 (100)	0 (0)
2011	2	2 (100)	0 (0)
2012	5	5 (100)	0 (0)
2013	2	1 (50)	1 (50)
2014	4	1 (25)	3 (75)
2015	5	1 (20)	4 (80)
2016	5	1 (20)	4 (80)
2017	5	2 (40)	3 (60)
2018	5	0 (0)	5 (100)
2019	5	0 (0)	5 (100)
2020	2	0 (0)	2 (100)
**Total**	**50**	**23 (46)**	**27 (54)**

## Data Availability

The consensus sequences of PCV-2 ORF2 were deposited at the NCBI GenBank with the numbers (MZ299032–MZ299081).
